# Treatment of electroencephalographic status epilepticus after cardiopulmonary resuscitation (TELSTAR): study protocol for a randomized controlled trial

**DOI:** 10.1186/1745-6215-15-433

**Published:** 2014-11-06

**Authors:** Barry J Ruijter, Michel JAM van Putten, Janneke Horn, Michiel J Blans, Albertus Beishuizen, Anne-Fleur van Rootselaar, Jeannette Hofmeijer, on behalf of the TELSTAR study group

**Affiliations:** Clinical Neurophysiology group, MIRA - Institute for Biomedical Technology and Technical Medicine, University of Twente, Hallenweg 15, 7522NB Enschede, The Netherlands; Departments of Neurology and Clinical Neurophysiology, Medisch Spectrum Twente, Haaksbergenstraat 55, 7513 ER Enschede, The Netherlands; Department of Intensive Care Medicine, Academic Medical Center, Meibergdreef 9, 1005 AZ Amsterdam, The Netherlands; Department of Intensive Care Medicine, Rijnstate Hospital, Wagnerlaan 55, 6815 AD Arnhem, The Netherlands; Department of Intensive Care Medicine, Medisch Spectrum Twente, Haaksbergenstraat 55, 7513ER Enschede, The Netherlands; Departments of Neurology and Clinical Neurophysiology, Academic Medical Center, Meibergdreef 9, 1005 AZ Amsterdam, The Netherlands; Department of Neurology, Rijnstate Hospital, Wagnerlaan 55, 6815 AD Arnhem, The Netherlands

**Keywords:** Cardiopulmonary resuscitation, Postanoxic encephalopathy, Electroencephalography, Status epilepticus, Generalized periodic discharges, Anticonvulsants, Randomized controlled trial, Neurological outcome

## Abstract

**Background:**

Electroencephalographic (EEG) status epilepticus is described in 10 to 35% of patients with postanoxic encephalopathy after successful cardiopulmonary resuscitation and is associated with case fatality rates of 90 to 100%. It is unclear whether these EEG patterns represent a condition to be treated with anticonvulsants to improve outcome, or an expression of severe ischemic damage, in which treatment is futile.

**Methods/Design:**

TELSTAR is a multicenter clinical trial with two parallel groups, randomized treatment allocation, open label treatment, and blinded endpoint evaluation (PROBE design). We aim to enroll 172 adult patients with postanoxic encephalopathy and electroencephalographic status epilepticus after successful cardiopulmonary resuscitation, admitted to the ICU, in whom continuous EEG monitoring is started within 24 hours after admission. Patients are randomly assigned to either medical treatment to suppress all electrographic seizure activity, or no treatment of electroencephalographic status epilepticus. Antiepileptic treatment is based on guidelines for treatment of overt status epilepticus and is started within 3 hours after the diagnosis. If status epilepticus returns during tapering of sedative medication after suppression of all epileptiform activity for 2 × 24 hours, it will be considered refractory. The primary outcome measure is neurological outcome defined as the Cerebral Performance Category (CPC) score at 3 months, dichotomized into ‘good’ (CPC 1 to 2 = no or moderate neurological disability) and ‘poor’ (CPC 3 to 5 = severe disability, coma, or death). Secondary outcome measures include mortality and, for patients surviving up to 12 months, cognitive functioning, health related quality of life, and depression.

**Trial registration:**

Clinicaltrials.gov; NCT02056236. Date of registration: 4 February 2014.

## Background

After successful cardiopulmonary resuscitation, 64 to 74% of patients remain unconscious at hospital arrival as a result of diffuse postanoxic encephalopathy [1, 2]. In these patients, a spectrum of electroencephalographic (EEG) changes can be observed, reflecting varying extents of ischemic brain injury [3]. Electroencephalographic status epilepticus is described in 10 to 35% [3–8] and is strongly associated with poor outcome: case fatality was 90 to 100% in prospective case series, despite treatment with anticonvulsants [4, 6–10]. Without EEG monitoring, approximately one in five cases remains undiscovered due to the absence of clinical signs [6, 11, 12].

The diagnostic criteria for electroencephalographic status epilepticus in comatose patients with postanoxic encephalopathy are controversial [13, 14]. The American Clinical Neurophysiology Society defines unequivocal seizures as generalized spike-wave discharges at 3 Hz or faster or clearly evolving discharges of any type at 4 Hz or faster, either focal or generalized [15]. However, some experts also consider other rhythmic or periodic patterns, such as generalized or lateralized periodic discharges or rhythmic delta activity as seizure activity [6, 8].

It is unclear whether electroencephalographic seizure patterns in patients with postanoxic encephalopathy represent a condition which can be treated with anticonvulsants to improve patients’ outcome, or have to be regarded as an expression of severe ischemic damage, in which treatment with anticonvulsants would be futile [16, 17]. Case series suggest that in patients with electroencephalographic status epilepticus, preserved brainstem reactions and EEG background reactivity are associated with a favorable outcome [4]. It is unclear whether treatment with anticonvulsants reduces the risk of a poor outcome in these patients and if so, how intensive this treatment should be. In the only prospective, non-randomized intervention study, a stepwise treatment up to pentobarbital-induced burst suppression resulted in a good outcome for 6% of patients with clinically overt or electroencephalographic status epilepticus [18]. This proportion is approximately the same as reported in observational studies, irrespective of treatment [4, 6, 8, 9].

Despite the lack of evidence, most neurologists treat status epilepticus in comatose patients after cardiopulmonary resuscitation with anticonvulsants. Increased detection of electroencephalographic status epilepticus by continuous EEG monitoring has led to increased prescription of these drugs [19, 20]. However, treatment is mostly moderately intensive. In The Netherlands, only one third of physicians treat these patients equally intensively as those with clinically overt status epilepticus [21]. Both intensive antiepileptic treatment and no treatment of electroencephalographic status epilepticus are considered standard modalities, where some experts believe that treatment is useless and others that it is unethical to withhold it.

Apart from the intensity, the timing of treatment is probably an important determinant of treatment effect. Mechanisms such as excessive glutamate release are known to worsen brain damage in ongoing status epilepticus within 20 to 40 minutes, even without clinical signs and despite good oxygenation [22]. Also, prolonged duration of status epilepticus reduces the effect of treatment; for example, due to receptor trafficking [23]. In approximately a quarter of patients, the electroencephalographic status epilepticus starts within 24 hours after cardiopulmonary resuscitation [5, 10, 12]. In previous studies, EEG monitoring started at a median of 2 to 3 days after cardiopulmonary resuscitation, indicating that diagnosis and subsequent treatment of electroencephalographic status epilepticus started relatively late [4, 8].

We conclude that evidence for a beneficial effect of medical treatment of electroencephalographic status epilepticus in patients with postanoxic encephalopathy after cardiopulmonary resuscitation is insufficient. To be effective, treatment should be sufficiently intensive and initiated as early as possible after the onset of status epilepticus. Therefore, we aim to study the effect of intensive and early medical treatment of electroencephalographic status epilepticus on functional outcome of comatose patients after cardiopulmonary resuscitation in a randomized controlled clinical trial.

### Hypothesis

Medical treatment of electroencephalographic status epilepticus improves outcome of patients with postanoxic encephalopathy after successful cardiopulmonary resuscitation.

## Methods/Design

### Design and population

TELSTAR is a multicenter clinical trial with two parallel groups, randomized treatment allocation, open label treatment and blinded endpoint evaluation (PROBE design). The trial has been registered in the United States National Institutes of Health Clinical Trials registry (clinicaltrials.gov, identifier NCT02056236) on 4 February 2014. The study population consists of comatose adult patients after cardiopulmonary resuscitation, admitted to the ICU, with electroencephalographic status epilepticus on continuous EEG. Detailed eligibility criteria are listed in Table 1.

**Table 1:** **Eligibility criteria**

Inclusion criteria	Exclusion criteria
Patients after successful cardiopulmonary resuscitation with suspected postanoxic encephalopathy	A known history of another medical condition with limited life expectancy (<6 months)
Age 18 years or older	Any progressive brain illness, such as a brain tumor or neurodegenerative disease
Continuous EEG with at least 8 electrodes started within 24 hours after cardiopulmonary resuscitation	Pre-admission glasgow outcome scale score of 3 or lower
Electroencephalographic status epilepticus on continuous EEG	Reason other than neurological condition to withdraw treatment
Informed consent given by a legal representative	Follow-up impossible due to logistic reasons, for example not living in The Netherlands
Possibility to start treatment within 3 hours after detection of electroencephalographic status epilepticus^a^	Known participation in any interventional study

^a^If status epilepticus is present at initiation of continuous EEG, the starting time of continuous EEG is considered as detection time. EEG = electroencephalography.

Definitions of electroencephalographic status epilepticus will be according to standardized critical care EEG terminology [15]. They may consist of generalized spike-wave discharges at 3 Hz or faster, clearly evolving discharges of any type at 4 Hz or faster (either generalized or focal), or periodic discharges (generalized or lateralized) at any frequency. For continuous seizure activity, the minimum duration requirement is 30 minutes. Intermittent seizures of 5 minutes and longer, recurring at least twice, with seizure-free intervals shorter than 60 minutes will also be included. EEG assessment for inclusion will ultimately be left to the discretion of the treating neurologist or clinical neurophysiologist.

### Informed consent

Written informed consent will be obtained from the patient's authorized representative prior to the performance of any protocol-specific procedure. Surviving patients will be asked for informed consent for participation and additional follow-up on long-term outcome. Separate informed consent will be asked for neuropsychological examination at 12 months, if applicable. The study will be conducted according to the principles of the Declaration of Helsinki (Seventh Revision, Fortaleza, 2013) and in accordance with the Dutch Medical Research Involving Human Subjects Act (WMO) and local guidelines.

### Treatment allocation

All participating subjects receive standard best medical management according to current guidelines, including therapeutic hypothermia or controlled normothermia with sedation for 24 hours. In addition, they will be randomly assigned to either medical treatment or no treatment of electroencephalographic status epilepticus. In both groups, decisions regarding limitation or withdrawal of treatment will be made in accordance with the Dutch guidelines for postanoxic coma management [24]. Reasons for withdrawal of treatment will be documented.

Subjects will be randomized using ALEA (Clinical Trial Center Maastricht, The Netherlands), which is an online, central randomization service. To prevent imbalance of allocated treatments, blocked randomization will be used, with a 1:1 allocation, stratified by center, and random block size ranging from 4 to 10 subjects.

### Intervention group: treatment of electroencephalographic status epilepticus

**Figure 1:**
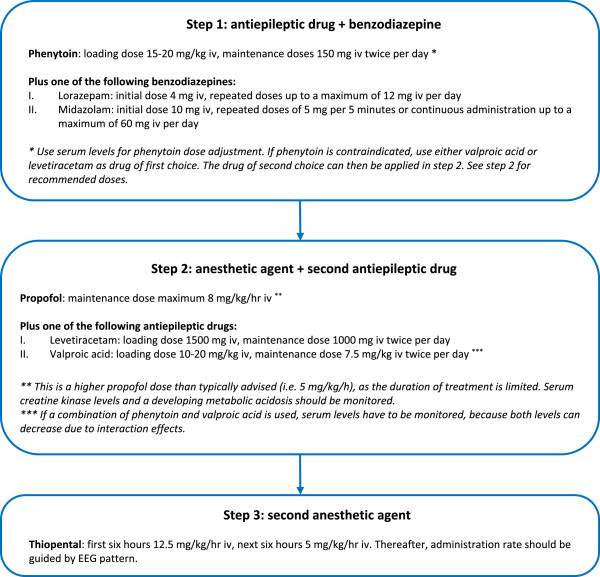
**Stepwise treatment approach for electroencephalographic status epilepticus.** Each consecutive treatment step is taken when previous steps have failed to suppress electroencephalographic seizure activity. After 24 hours of treatment, sedative agents (thiopental, propofol, and continuously administrated benzodiazepines) will be tapered and intermittently administrated antiepileptic drugs (phenytoin, valproic acid, and levetiracetam) will be continued. If the status epilepticus returns, the treatment procedure will be repeated during another 24 hours. If status epilepticus returns after 2 × 24 hours, it is considered refractory. EEG = Electroencephalography.

Since no treatment with anticonvulsants has been proven superior to another, the choice of medication is ultimately left to the discretion of the treating neurologist, based on local protocols. However, to prevent large differences with respect to the intensity of treatment, recommendations are made as displayed in Figure 1. In summary, treatment consists of a stepwise approach, from intermittent administration of antiepileptic drugs up to treatment with barbiturates, in accordance with international guidelines for treatment of overt status epilepticus [25, 26]. Each subsequent step is taken as soon as possible when previous steps fail to suppress epileptiform activity. The treatment objective is to suppress all epileptiform activity on the EEG during at least 24 hours. Induction of burst suppression is not obligate. If the status epilepticus returns after tapering sedative medication, the treatment procedure will be repeated during another 24 hours. If status epilepticus returns after 2 × 24 hours, it is considered refractory. We accept that treatment with barbiturates may lead to prolonged hospitalization of several days due to their influence on prognostic tests.

### Control group: no treatment of electroencephalographic status epilepticus

In this group, patients will be treated according to standard guidelines for treatment of comatose patients after cardiopulmonary resuscitation, without EEG-based treatment of status epilepticus. Treatment to suppress clinically manifest myoclonic jerks in this group, and in those patients surviving beyond ICU discharge, is left to the discretion of the treating physician.

### Outcome assessment

The primary outcome measure is neurological outcome, defined as the Cerebral Performance Category (CPC) score at 3 months, dichotomized into ‘good’ (CPC 1 to 2 = no or moderate neurological disability) and ‘poor’ (CPC 3 to 5 = severe disability, coma, or death).

Secondary outcome measures include mortality, CPC scores at 6 and 12 months, length of stay on the ICU, duration of mechanical ventilation, and seizure recurrence rate within 12 months. In case of survival, additional outcome measures include quality of life after a year as measured by the Medical Outcomes Study 36-item short-form health survey (SF-36) [27], depression after a year as measured by the Montgomery and Åsberg Depression Rating Scale (MADRS) [28], and cognitive functioning after a year as measured by detailed neuropsychological examination. Secondary outcome measures will not be collected to test between-group differences, since the estimated number of survivors is small. These measures will be collected to thoroughly assess outcome and quality of life of survivors.

Additionally, a limited amount of data on the use of resources will be collected for analysis of cost-effectiveness, including place of residence at one year and admission to hospitals, rehabilitation centers, and nursing homes within the first year.

### Study procedures and data collection

For data collection and management, the OpenClinica open source software (OpenClinica LLC and collaborators, Waltham, MA, USA) will be used. At randomization, patients receive a study number by which all data is coded. The trial coordinator and principal investigator safeguard the key to this code.

**Figure 2:**
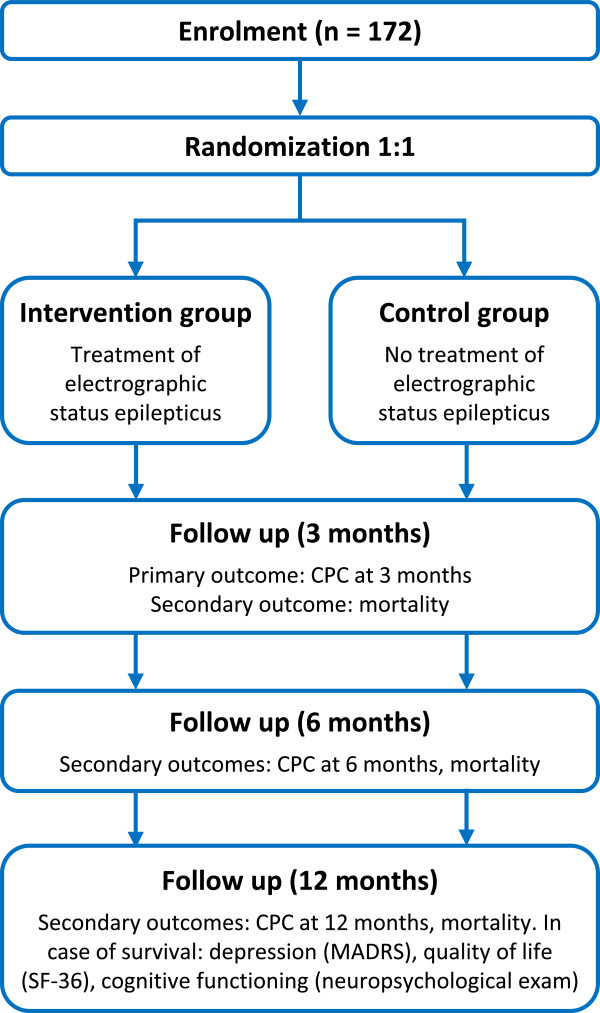
**Study flow chart.** CPC = Cerebral Performance Category; MADRS: Montgomery and Åsberg Depression Rating Scale; SF-36: Medical Outcomes Study 36-item short-form health survey.

Figure 2 shows the study flow chart. In participating hospitals, continuous EEG monitoring is part of regular patient care and is initiated as soon as possible after admission to the ICU. In order to detect seizure activity early, continuous EEG registrations are checked three-hourly by a neurologist, clinical neurophysiologist, or clinical neurophysiology technician. For practical reasons, these checks will not take place regularly between midnight and 8 am. When patients meet the eligibility criteria and informed consent is obtained, they will be randomly allocated to one of the treatment arms. It is not possible to see the treatment allocation before the patient is randomized and registered in the study database. Neither is it possible to withdraw the patient from the database after treatment assignment. After randomization, treating physicians and patients (or their legal representatives) will be aware of the treatment assignment.

Baseline data will be obtained at randomization and include patient characteristics (age, sex, medical history, use of anticonvulsants), pre-hospital factors (cause and location of cardiac arrest, initial cardiac rhythm, time to return of spontaneous circulation), clinical parameters (Glasgow Coma Scale score, brain stem reflexes), electroencephalographic features (seizure pattern, background reactivity), target temperature, and the Acute Physiology and Chronic Health Evaluation (APACHE) score. During admission on the ICU, daily neurological examinations will be performed as part of regular care. Selected medication for treatment of the status epilepticus will be recorded. At ICU discharge or in case of mortality, the duration of ventilation and duration of admission will be assessed.

Follow-up will be done by means of a telephone interview by a trained investigator or research nurse, who is blinded for the allocated treatment. At 3, 6, and 12 months, CPC scores will be assessed. Place of residence, duration of admission in hospitals, rehabilitations centers, or nursing homes, seizure recurrence rates, use of anticonvulsants, and serious adverse events will be recorded. At 12 months, after obtaining separate informed consent, quality of life, depression, and cognitive functioning will be assessed in the local hospital.

Subjects can leave the study at any time for any reason if they wish to do so without any consequences. Also, the investigator can decide to withdraw a subject from the study for urgent medical reasons. Subjects will be replaced after withdrawal for any reason. Every attempt will be made to complete the primary follow-up in patients who are withdrawn from treatment.

### Statistical considerations

The primary analysis will be a single comparison between the treatment groups with regard to the primary outcome measure at 3 months, according to the intention-to-treat principle. To assess the effect of treatment with anticonvulsants, the absolute risk reduction of poor outcome will be calculated, including the corresponding 95% confidence interval. Baseline characteristics, raw distributions of the CPC score, and scores of secondary outcome measures will first be presented in a descriptive way. For secondary outcome measures, between-group differences will be analyzed by means of independent samples *t*-tests, Mann–Whitney tests, or Fisher exact tests, where appropriate. If necessary, multivariate regression analysis will be used to adjust for imbalances in main prognostic variables between the intervention and control groups.

Power calculations are hampered by the absence of data from randomized trials. With a presumed reduction of poor outcome from 99% to 92%, alpha of 5%, power of 80%, and 1-tailed testing, 84 patients per treatment group are needed to detect superiority of treatment with anticonvulsants. An O’Brien Fleming interim analysis will be performed after a total of 86 enrollments. If the difference between the treatment groups at that time is significant at *P* <0.00557, the trial will be stopped because of ‘proof beyond reasonable doubt’ that treatment with anticonvulsants is superior to treatment without anticonvulsants. To compensate for this interim analysis, two additional patients per group will be included. This indicates an intended sample size of 172 subjects.

With an estimated incidence of electroencephalographic status epilepticus of 20% in comatose patients with postanoxic encephalopathy [3], 860 patients will have to be monitored with continuous EEG. With five Dutch hospitals participating at the submission of this manuscript, we estimate an enrollment period of four and a half years.

### Safety assessments

Medical treatment of electroencephalographic status epilepticus may lower the high risk of death. The risk of an increase of morbidity or mortality is considered negligible. Otherwise, treatment may lead to prolonged hospitalization of several days of comatose patients who otherwise would have died. We assume this reasonable, since both antiepileptic treatment and no treatment are current standard modalities in these patients.

All adverse events reported by the investigator, staff, or spontaneously by the subject will be recorded in the electronic case record form. Adverse events are defined as any undesirable experience occurring to a subject during the clinical trial. A serious adverse event is defined as any adverse event that results in death, a life-threatening condition, inpatient hospitalization or prolongation of existing hospitalization, or persistent or significant disability.

The trial is monitored by an independent Data Safety Monitoring Board (DSMB). The DSMB is chaired by a biostatistician and further includes an intensivist and a neurologist. This committee will review data for safety after every 43 enrolled patients from the study have had their primary outcome measurement. Because of the expected high proportion of patients with a poor outcome in the study population, and the consequent overlap between safety endpoints and the primary endpoint, the evaluation of safety by the DSMB at that time will be qualitative. Summary of key efficacy endpoints (primary outcomes, mortality) will be provided for a planned interim analysis after 86 enrollments.

### Ethical approval

TELSTAR has received ethical approval from the Medical Research Ethics Committee Twente (Dutch: METC Twente) in December 2013 (reference: NL46296.044.13).

### Publication of trial results

The trial results will be published by the members of the Executive Committee, on behalf of the TELSTAR study team. With 36 or more enrollments, local principle investigators of participating centers will be included in the primary author list. Before submission of any manuscript, all local principal investigators will have had the opportunity to comment on the manuscript.

## Discussion

We present a study protocol for a multicenter, randomized controlled trial to investigate treatment of electroencephalographic status epilepticus in patients with postanoxic encephalopathy after cardiopulmonary resuscitation. Both sides of the spectrum, for example, antiepileptic treatment to burst suppression and no treatment at all of electroencephalographic status epilepticus are current standard modalities in these patients [19, 21]. The benefits of antiepileptic treatment have never been investigated in a randomized controlled trial.

The focus of debate is often whether EEG patterns represent epileptic activity, that can be treated with anticonvulsants to improve outcome, or not [13, 14]. In the only prospective case series of intensive antiepileptic treatment in these patients, EEG patterns of enrolled subjects were insufficiently reported [18]. In the current trial, we include a broad range of possibly epileptiform EEG patterns. However, with a clear definition of the distinct eligible seizure patterns, based on standardized EEG terminology [15], we aim to increase insight into the effects of treatment in relation to specific patterns. Although EEG analysis prior to patient enrollment will be performed locally by the treating neurologist or clinical neurophysiologist, all EEGs will also be analyzed off-line by dedicated clinical neurophysiologists, to optimize categorization of patterns.

If beneficial at all, the optimal duration of treatment of status epilepticus in postanoxic encephalopathy is unclear. Previous studies suggest that in more than half of cases the duration of seizure activity is less than 24 hours, and in more than three quarters of cases less than 48 hours [5, 12]. We consider ongoing status epilepticus after suppression of all epileptiform activity for 2 × 24 hours as refractory and argue that patients with ongoing seizure activity beyond this period are unlikely to benefit from prolonged antiepileptic treatment.

In previous, non-randomized studies concerning treatment of status epilepticus after cardiopulmonary resuscitation, diagnosis was either based on clinical signs [29–31], or EEG seizure activity [6, 8, 10], or a combination of clinical and EEG findings [4, 18]. However, myoclonic jerks are often suppressed by sedative medication. Furthermore, myoclonia are often unrelated to EEG findings. For most neurologists, the threshold to treat patients with overt myoclonia is lower than for patients with non-convulsive electroencephalographic seizures. However, irreversible damage is probably even more likely in patients with myoclonia, since the risk of poor outcome is larger [4] and neuronal necrosis is more common [17]. In the current study, the diagnosis of status epilepticus is EEG-based, irrespective of clinical signs.

Our study protocol intends to minimize time from onset of electroencephalographic status epilepticus to start of treatment, as ongoing seizure activity may lead to additional damage [22] and decreased effect of medication [23]. EEG monitoring will be started within 24 hours. Epileptic activity that is initially suppressed by the anticonvulsive effects of hypothermia and sedative medication will therefore be detected early. The maximum time of 3 hours since the diagnosis to obtain informed consent and commence treatment is chosen pragmatically.

Although the trial protocol contains clear recommendations, including the intensity of antiepileptic treatment in the intervention group, the diagnosis of electrographic status epilepticus and the choice of specific anticonvulsive medication are ultimately left to the discretion of the treating physician. Since we consider the pragmatic character of this difficult trial very important, we accept potentially detrimental effects of variations due to freedom of treatment. These include restricted extrapolation resulting from differences between local protocols and effect dilution resulting from treatment of overt myoclonia with anticonvulsants in the control group.

Blinding of treating physicians for treatment allocation is virtually impossible, as the administration of anticonvulsants is guided by bedside EEG measurements. However, outcome assessment of surviving patients is performed blinded for the treatment allocation.

Concluding, this study will be the first to evaluate the additional value of medical treatment of electroencephalographic status epilepticus for patients after successful cardiopulmonary resuscitation. Furthermore, it will provide clinical and electrographic characteristics for identifying patients that might benefit from this treatment.

## Trial status

Recruitment to TELSTAR started in May 2014, with 5 participating hospitals. Interested centers with ICU and continuous EEG monitoring facilities are welcome to participate.

## Abbreviations

APACHE: Acute Physiology and Chronic Health Evaluation
CPC: Cerebral Performance Category
DSMB: Data Safety Monitoring Board
EEG: electroencephalography
MADRS: Montgomery and Åsberg Depression Rating Scale
PROBE: Prospective Randomized Open Blinded Endpoint
SF-36: Medical Outcomes Study 36-item short-form health survey
WMO: Dutch Medical Research Involving Human Subjects Act.
